# Characterization of anti-AAV2 neutralizing antibody levels in sheep prior to and following intravitreal AAV2.7m8 injection

**DOI:** 10.1038/s41434-024-00495-5

**Published:** 2024-10-29

**Authors:** Maya Ross, Kareen Sade, Alexey Obolensky, Edward Averbukh, Melissa Desrosiers, Alexander Rosov, Hay Dvir, Elisha Gootwine, Eyal Banin, Deniz Dalkara, Ron Ofri

**Affiliations:** 1 Department of Animal Science, ARO, The Volcani Center, Rishon LeZion, Israel; 2 Koret School of Veterinary Medicine, Hebrew University of Jerusalem, Rehovot, Israel; 3 Department of Ophthalmology, Hadassah Medical Center, Faculty of Medicine, Hebrew University of Jerusalem, Jerusalem, Israel; 4 Sorbonne Université, INSERM, CNRS, Institut de la Vision, 17 rue Moreau, F-75012 Paris, France

**Keywords:** Immunological disorders, Neurological disorders

## Abstract

Gene augmentation therapy is a promising treatment for incurable, blinding inherited retinal diseases, and intravitreal delivery is being studied as a safe alternative to subretinal injections. Adeno-Associated Viruses (AAV) are commonly-used vectors for ocular gene augmentation therapy. Naturally occurring pre-operative exposure and infection with AAV could result in presence of neutralizing antibodies (NAB’s) in patients’ serum, and may affect the safety and efficacy of treatment. Our aim was to characterize the humoral response against AAV pre- and post-intravitreal delivery of AAV2.7m8 vectors in a naturally-occurring sheep model of *CNGA3* achromatopsia. Serial serum neutralization assays were performed to screen sheep for pre-exiting anti-AAV2 NAB’s, and to assess the effect of intravitreal AAV2.7m8 injection on post-operative NAB titers and intraocular inflammation in sheep. The effect of viral dose and transgene type were also assessed. Serological screening revealed pre-operative seropositivity in 21.4% of animals, with age being a risk factor for the presence of anti-AAV2 NAB’s. NAB titers increased following intravitreal AAV administration in the majority of sheep. There was no significant difference in the degree of post-operative serum neutralization between pre-operatively seronegative sheep and those with pre-existing antibodies. However, only sheep with pre-existing antibodies presented with signs of post-operative inflammation. We conclude that pre-existing anti-AAV2 NAB’s do not affect the level of post-operative NAB titers; however, they increase the risk of post-operative ocular inflammation. Our results could have implications for the management of AAV-mediated ocular gene therapies, a technology being increasingly studied and used in patients.

## Introduction

Gene augmentation therapy for inherited retinal diseases is an emerging field, with a large number of pre-clinical and clinical trials being completed worldwide with the aim of developing treatments for incurable blinding diseases [1]. Currently, the most commonly used viral vector for gene augmentation therapy are different serotypes of recombinant Adeno-Associated Virus (rAAV) [2]. rAAV is derived from the nonpathogenic AAV virus of the *Parvoviridae* family. Wild-type AAVs contain a linear single-stranded DNA genome of ~4.7 kb enclosed within a capsid. Recombinant AAV vectors are devoid of all viral genes encoding replication and capsid proteins and are packed with a desired transgene [3]. rAAVs are considered good candidates for gene augmentation therapy because of their lack of pathogenicity, replication and cytotoxicity, low immunogenicity, and long-term transgene expression following a single treatment [4]. An important limitation of using rAAVs is their maximal packaging capacity of approximately 5.0 kb [2].

A significant barrier to effective intraocular rAAV gene delivery is the interaction of the vector with the host’s immune system [5]. A primary immune response to the treatment may be triggered by the presence of pre-existing neutralizing antibodies (NABs) against rAAV capsids, due to previous naturally-occurring exposure to AAV [1, 3]. NABs are a subset of anti-AAV antibodies that bind to the virus and prevent cellular transduction and transgene expression. A significant titer of anti-AAV antibodies was found in primates and the presence of pre-existing antibodies has been correlated with decreased efficacy of gene augmentation therapy in pre-clinical trials where the route of administration exposes vector to serum [6]. Serology studies in humans revealed high pre-operative titers of anti-AAV antibodies for AAV2 (72%), AAV9 (47%) and other AAV serotypes [7]. The presence of these pre-existing antibodies in candidates for ocular gene augmentation therapy may reduce treatment efficacy due to neutralization of the virus, and because of the induced immune-mediated inflammation [8]. Therefore, screening of experimental animals and potential patients for the presence of anti-AAV NABs prior to intraocular delivery should be considered [9].

Several other factors can impact the host’s immune response to intraocular rAAV delivery, and therefore affect the degree of cellular transduction, efficacy and safety of retinal gene augmentation therapy. First is the mode of delivery (subretinal [SR] or intravitreal [IVT] injections), as some studies demonstrate that the subretinal space is more immune privileged than the vitreous [10–13], though this finding has been disputed by others [8]. Second, the total dose injected was shown to have the largest effect both on treatment efficacy and on the degree of immune response, with higher viral doses resulting in increased levels of serum antibodies post-injection [8]. Finally, the type of promoter [14], AAV serotype [15] and type of transgene [16] also affect treatment outcome and the degree of post-operative inflammation.

For the past 12 years, our group has been studying day blindness in sheep, and using this naturally-occurring large animal model to develop gene augmentation therapy for achromatopsia patients. Sheep in these studies are homozygous for one of two mutations in the *CNGA3* gene, encoding for a subunit of the cyclic nucleotide gated channel in cone photoreceptors. SR injection of rAAV2 carrying the *CNGA3* gene successfully restored sheep’s photopic vision with a long-lasting effect [17–19], contributing to approval of clinical trials in *CNGA3* achromatopsia patients (NCT02935517, NCT02610582, NCT03278873).

However, despite promising results of SR-based gene augmentation therapy in our sheep model and in numerous other studies, this delivery route suffers from several drawbacks; it causes a localized retinal detachment, it only transduces a limited portion of the retina and it must be performed under general anesthesia, using advanced surgical techniques and equipment, leading to higher costs and potential risks and complications [1, 20]. IVT delivery may be a promising alternative route of vector delivery as it does not create a retinal detachment, it has the potential to transfect the entire retina and it is a safe procedure performed routinely on outpatients using only topical anesthesia. However, compared to SR delivery, IVT injections may trigger a more robust immune response against the rAAV vector because of its closer proximity to the vascular system, the larger doses required to compensaet for dilution in the vitreous, and the lack of a blood retinal barrier that exists in the SR but not in the IVT space [6].

Due to the potential advantages of IVT injections, in recent years we have studied the safety and efficacy of this delivery method in our sheep model [14, 21]. As part of these studies, serum samples were collected from sheep before and after treatment and stored for future analysis, because a high level of naturally occurring anti-AAV2 antibodies was previously reported in sheep [22]. The purpose of this retrospective study was to characterize and evaluate the humoral response against AAV2 in sheep pre- and post-IVT rAAV2 delivery. We therefore analyzed the titers of NABs in serum samples collected over the years from sheep of the experimental Volcani Center herd prior to IVT injection, and at several time points following injection. An NAB assay was used to determine whether sheep in the experimental herd have naturally occurring anti-AAV antibodies before treatment; whether IVT injections cause an elevation in anti-AAV NAB titers and clinical inflammation; and whether the post-injection NAB titers and inflammation are affected by the presence of pre-existing antibodies. We demonstrate that pre-existing anti-AAV2 NAB’s do not affect the level of post-operative NAB titers in treated sheep; however, they result in more pronounced ocular inflammation post-operatively.

## Materials and Methods

### Animals and surgical procedures

Experimental protocols were approved by the Volcani Center Animal Experimentation Ethics Committee and were conducted in accordance with the Association for Research in Vision and Ophthalmology’s Statement for Use of Animals in Ophthalmic and Vision Research. A serological survey was conducted in seventy (63 wild type and 7 day blind) ewes randomly chosen from the experimental flock of Volcani Center that are kept in an outdoor pen. Sample size was calculated using WinPepi (V 11.65, given a population of 300 sheep and an assumed rate of 17 cases per 100). From this cohort, 12 ewes (including 3 day blind sheep) were chosen to undergo IVT treatment. All 12 sheep were found to be systemically healthy following a comprehensive physical examination by a board-certified specialist in small ruminant medicine, and ophthalmologically healthy (except day blindness in 3 animals) following a comprehensive ophthalmic examination by a board-certified veterinary ophthalmologist (below). For IVT injections, animals were pre-medicated with an intramuscular injection of pethidine (3 mg/kg; Dolestine, Teva Pharmaceutical Industries, Israel) and acepromazine (0.1 mg/kg; 10 mg/ml compounded preparation, Vetmarket Pharmacy, Israel). Pupils were dilated with 0.5% tropicamide (Mydramide, Fischer Pharmaceutical Labs, Israel) and 10% epinephrine (Efrin-10, Fischer Pharmaceutical Labs, Israel). Anesthesia was induced with an intravenous injection of propofol (4 mg/kg; Propofol-lipuro, B. Braun Medical Supplies, Philippines) and diazepam (0.15 mg/kg; Assival, Teva Pharmaceutical Industries, Israel) and maintained with 2–3% isoflurane (Forane, Abbott Laboratories, England). Eyes were surgically scrubbed, and injections conducted as previously described [14, 21]. Briefly, a single 23 G pars plana port was created in the ventral aspect of the globe, 2 mm from the limbus. A 41 G retractable subretinal injection needle (DORC 1270.ext, Dutch Ophthalmic Research Center, Zuidland, Netherlands), attached to a short extension line and a 1 ml syringe containing the viral vector was introduced through the port, and the viral vector was injected into the vitreous. The port was removed, paracentesis of the anterior chamber to reduce intraocular pressure was performed when indicated, and eyes were injected subconjunctivally with 2 mg cefuroxime and 2 mg dexamethasone. Following surgery, animals were treated with a topical neomycin and dexamethasone solution (Dethamycin, Vitamed Ltd, Binyamina, Israel) three times daily for one week. The vector, promoter and transgene injected and the inoculum volume and vector dose used in each animal are detailed in Table 1.

**Table 1:** Sheep, pre- and post-operative serological status, vector, promoter, transgene, volumes and doses injected in the study cohort.

Sheep	Age at 1^st^ sampling (months)	Pre-op serological status (neutralizing dilution)	Uni/bi lateral treatment	Vector-promoter- transgene	Total vg injected (inoculum volume)	12 weeks post-op serological status (neutralizing dilution)
9402	0.87	Negative	Bi	AAV2-7m8-CAG-eGFP	4.00E + 12 (400 µl)	Positive (1/2560)
9349	1.07	Negative	Bi	AAV2-7m8-CAG-eGFP	3.00E + 12 (400 µl)	Positive (1/160)
9399	0.87	Negative	Bi	AAV2-7m8-PR.1.7-eGFP	3.00E + 12 (300 µl)	Positive (1/160)
9364	1.03	Negative	Bi	AAV2-7m8-PR.1.7-eGFP	3.00E + 12 (400 µl)	Negative
9384	0.93	Negative	Bi	AAV2-7m8-PR.1.7-eGFP	2.50E + 12 (900 µl)	Positive (1/1280)
9277	16.87	Negative	Uni	AAV2-7m8-PR.1.7-hCNGA3	3.38E + 12 (325 µl)	Positive (1/40)
8967	20.6	Negative	Uni	AAV2-7m8-PR.1.7-hCNGA3	3.38E + 12 (325 µl)	Positive (1/40)
5789	104.1	Positive (1/40)	Uni	AAV2-7m8-non-coding	1.50E + 11 (150 µl)	Positive (1/40)
8495	69.87	Positive (1/40)	Uni	AAV2-7m8-non-coding	1.50E + 11 (150 µl)	Positive (1/40)
8946	62.4	Positive (1/640)	Uni	AAV2-7m8-non-coding	1.50E + 11 (150 µl)	Positive (1/1280)
7759	85.7	Positive (1/160)	Uni	AAV2-7m8-non-coding	1.50E + 11 (150 µl)	Positive (1/2560)
8730	24.1	Positive (1/40)	Uni	AAV2-7m8-PR.1.7-hCNGA3	2.10E + 11 (450 µl)	Positive (1/1280)

*vg* viral genomes, *WT* wild type, *PR1.7* red/green opsin promoter (cone specific), *CAG* chicken beta-actin promoter (ubiquitous), *eGFP* enhanced green fluorescent protein, *hCNGA3* human cyclic nucleotide gated channel subunit A3.

In animals injected bilaterally, numbers in the “total vg injected (inoculum volume)” column are the volume and total dose injected into both eyes.

### Serum collection

Pre-operative serological screening for pre-existing neutralizing anti-AAV2 antibodies was routinely performed in sheep prior to participation in ongoing gene augmentation therapy experiments [17–19]. Seronegative animals were enrolled as cohorts 1 and 2 in a gene augmentation therapy study, the results of which were recently published [21]. Serum was collected post-operatively every 4 weeks for 12–16 weeks and kept frozen (−80 ^o^C) for future analysis. Seropositive animals were recruited for the current safety trial in which IVT injection of a non-coding vector was performed. Serum samples were collected four days, and two, four and twelve weeks post-operatively and kept frozen for analysis. Serum was also collected every four weeks for 12 weeks from one seronegative sheep that did not undergo any procedure, and served as a negative control.

### Serum neutralization assay

Serum neutralization assay was performed as previously described [9]. Briefly, HEK293T cells (American Type Culture Collection, Manassas, VA, USA) were cultured in Dulbecco’s Modified Eagle’s Medium (DMEM) supplemented with 10% fetal bovine serum and 1% penicillin/streptomycin (both from Biological Industries, Beit Haemek, Israel) at 37 °C and 5% CO_2_. Cells were seeded in 96-well plates at 70,000 cells/well in 100 μL of DMEM containing 10% fetal bovine serum and incubated overnight at 37 °C and 5% CO_2_. The following day, a series of sheep sera dilutions was prepared, and pooled sheep sera from sheep previously exposed to AAV were diluted in the same fashion and served as a positive control on all plates. AAV2.7m8 encoding the luciferase gene (AAV2-CMV-luciferase) was diluted to a concentration of 4.5 × 10^8^ vg. Next, 40 μL of the diluted AAV vector were mixed with 40 μL of each of the diluted sheep serum samples and incubated for 2 hr at 4 °C. From each mixture 25 μL were then transferred to an individual well of the cell culture plate in duplicates and the plate was incubated overnight at 37 °C and 5% CO_2_. The next morning wells were washed with PBS and luciferase activity was detected using the Luciferase Assay System (Promega, Madison, WI, USA). Briefly, cells were lysed with 20 μL of lysis buffer, followed by addition of 100 μL luciferase assay substrate to detect luminescence. Luminescence was detected using the Spectramax i3 luminometer (Molecular Devices, San Jose, CA, USA). Each plate also contained three wells with no sheep serum that were translated to 100% luminescence, and three additional wells with no virus that provided the background luminescence reading. Serum samples that reduced luminescence below 50% were considered to have neutralizing antibodies (seropositivity).

### Clinical ophthalmic examination

A board-certified veterinary ophthalmologist (RO) conducted comprehensive ophthalmic examinations, including slit lamp biomicroscopy and indirect ophthalmoscopy following pupillary dilation. All animals were examined pre-operatively, and then three to four and seven to ten days post-operatively. A third examination was performed four weeks post operatively in the seropositive group. Animals were not sedated for the examination. Clinical findings were categorized as either inflammatory (e.g., vitreous haze, retinal petechia) or secondary to the injection (e.g., subconjunctival hematoma or a focal cataract due to accidental striking of the lens). Inflammatory lesions were further graded on a scale of 0 – 4, as follows: 0 – no abnormalities detected; 1 – focal retinal petechia or vitreous hemorrhage; 2 – signs of mild anterior or posterior uveitis; 3 - focal retinal atrophy or detachment; 4 – significant uveitis or widespread ( > 15%) retinal atrophy or detachment. Lesions stemming from the procedure were graded as 0 and were not analyzed statistically.

### Statistical analysis

Statistical analysis was conducted using JMP® Pro 16.0.0 (SAS institute Inc., 2016. Cary, NC) and GraphPad Prism version 5.0. Sample size was calculated using WinPepi (V 11.65). The Shapiro-Wilk test was used to confirm normal distribution of the data. Student’s t test (two tailed, equal variance) was used to test for differences between groups of animals (pre-operative seropositive vs. pre-operative seronegative). Repeated measures ANOVA was used to analyze the effects of time and pre-operative serological status on post-operative NAB titers. The effect of viral vector dose on post-operative NAB titers was assessed by linear regression. A *p*-value below 0.05 was considered significant. As this was a retrospective study, the investigator was not blinded to the group allocation during the experiment and when assessing the outcome.

## Results

### Pre-existing immunity to AAV2 in sheep of the experimental herd

Seventy sheep from the experimental flock of the Volcani Center were serologically screened pre-operatively for pre-existing anti-AAV2 NABs. Fifteen sheep (21.4%) were found to be seropositive. Among them, six had low NAB titers (with 50% AAV neutralization obtained at a 40-fold serum dilution), seven had moderate NAB titers (with 50% AAV neutralization obtained at 160- or 640-fold serum dilutions) and two had high titers (with 50% AAV neutralization obtained at a 2560-fold serum dilution). The mean ± SE age of screened sheep was 16.7 ± 3.2 months (median 4.6 months). The mean age of the seronegative group (9.9 ± 2.6 months, median 2.8 months) was significantly lower than that of the seropositive group (41.8 ± 9.2 months, median 30.9 months, *p* = 0.004). In sheep older than one year of age seropositivity was 55% (11 seropositive sheep of 20 tested), compared to only 8% in sheep younger than one year (four seropositive sheep of 50 tested), with a 6.875 relative risk of seropositivity in sheep older than one year of age.

### Changes in NAB titers following IVT injection of AAV2.7m8

**Fig. 1:**
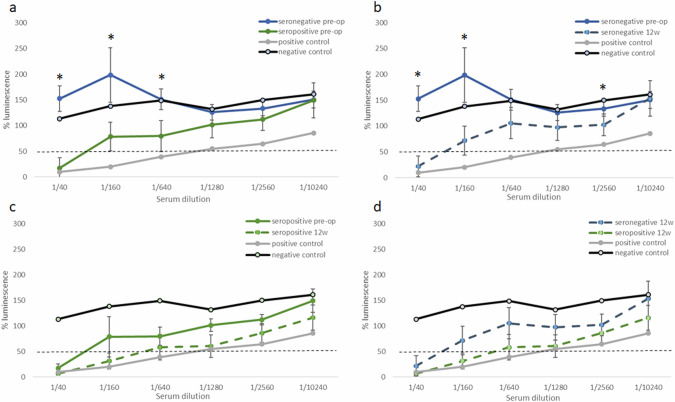
The degree of serum neutralization in sheep before, and 12 weeks after, IVT injection. NABs were tested by NAB serum neutralization assay. The graphs present the degree of luminescence (indicative of cellular transduction by AAV2) and its inhibition by NABs in serial dilutions of sheep serum. The values are shown as mean ± SE. Samples with no sheep serum were translated to 100% luminescence. Serum samples that reduced luminescence below 50% (dashed, horizontal line) were considered to have NABs. Two controls are depicted in each graph - a positive control of pooled serum from sheep who were previously exposed to AAV (gray), and a negative control of a seronegative sheep that did not undergo IVT injection (black). **a** Titers of anti-AAV2 NABs in seronegative (blue, *n* = 7) and seropositive (green, *n* = 5) sheep before IVT injection. **b** Change in the titers of anti-AAV2 NABs in seronegative sheep (*n* = 7), before (blue) and 12 weeks after IVT injection (dashed blue). **c** Change in the titers of anti-AAV2 NABs in seropositive sheep (*n* = 5), before (green) and 12 weeks after (dashed green) IVT injection. **d** Titers of anti-AAV2 NABs in seronegative (dashed blue, *n* = 7) and seropositive (dashed green, *n* = 5) sheep 12 weeks after IVT injection. Difference between pre- and post-operative samples (**b**, **c**) was tested using repeated measures ANOVA and difference between seropositive and seronegative sheep (panels a, d) was tested using Student’s t-test. *p < 0.05.

Of the 70 screened animals, twelve animals underwent uni- or bilateral IVT injection of rAAV2.7m8 followed by periodic serological testing and clinical evaluation. Seven sheep did not possess anti-AAV2 NABs prior to injection (seronegative) and were used in a recent gene augmentation therapy study [21]. Five other sheep had varying titers of pre-existing neutralizing antibodies (seropositive), and were used in the current safety trial. Of the seropositive animals used in this trial, three had low NAB titers and two had moderate NAB titers (Table 1). There was a significant difference in the degree of serum neutralization between the seronegative and seropositive groups prior to ocular injection (Fig. 1a). There was a significant increase of NAB titers in the seronegative group 12 weeks post-operatively (Fig. 1b), with six of the seven treated sheep developing NABs following IVT injection (Table 1). Three of the five seropositive sheep also had elevated NAB titers following IVT injection (Table 1); however, the average serum neutralization of the seropositive group was not significantly elevated post-operatively (Fig. 1c). There was no significant difference in NAB titers between the seronegative and seropositive groups 12 weeks post-operatively (Fig. 1d). There was no correlation between the total dose of viral vector injected and the degree of serum neutralization in sheep 12 weeks post-operatively.

### Clinical ophthalmic examination

Pre- and serial post-operative ophthalmic examinations were performed on all operated sheep. No abnormalities were detected in any of the eyes pre-operatively. Table 2 presents clinical findings that are potential immune responses and graded on a scale of 0–4. Three to four days post-operatively, mild, potentially immune-mediated clinical signs were observed in one sheep from the seropositive group. At the following ophthalmic exam, performed 7–10 days post-operatively, three sheep presented with mild to moderate clinical signs, all from the seropositive group. Statistical analysis revealed a significant difference between the grading of inflammation of the seropositive and seronegative groups on days 7–10 (*p* = 0.032). The seropositive sheep underwent a third examination, four weeks post-operatively, in which two sheep still presented with mild signs of inflammation. A third examination was not conducted in the seronegative animals, as there were no significant inflammatory findings in the previous examinations of this group.

**Table 2:** Sheep, Pre-operative serological status, post-operative clinical findings and clinical grading.

Sheep	Pre-op serological status (neutralizing dilution)	1st post-operative ophthalmic examination		2nd post-operative ophthalmic examination		3rd post-operative ophthalmic examination	
9402	Negative	No visible lesions	0	No visible lesions	0	N/A	
9349	Negative	No visible lesions	0	No visible lesions	0	N/A	
9399	Negative	Non-inflammatory lesions	0	Non-inflammatory lesions	0	N/A	
9364	Negative	No visible lesions	0	No visible lesions	0	N/A	
9384	Negative	No visible lesions	0	No visible lesions	0	N/A	
9277	Negative	Non-inflammatory lesions	0	No visible lesions	0	N/A	
8967	Negative	Non-inflammatory lesions	0	No visible lesions	0	N/A	
5789	Positive (1/40)	Two retinal petechiae and retinal edema in the injected eye), retinal petechiae in the fellow eye	2	Hyper-reflective area adjacent to ONH, intra-retinal bleeding in injected eye and fellow eye	3	Retinal petechiae in both eyes	1
8495	Positive (1/40)	Non-inflammatory lesions	0	Non-inflammatory lesions	0	No visible lesions	0
8946	Positive (1/640)	Non-inflammatory lesions	0	Mild vitreous haze, linear vitreal opacity lined with petechiae in the injected eye	2	Significant vitreal hemorrhage	2
7759	Positive (1/160)	Non-inflammatory lesions	0	Mild vitreous haze, linear vitreal opacity, with no evidence of blood	1	No visible lesions	0
8730	Positive (1/40)	Non-inflammatory lesions	0	No visible lesions	0	N/A	

All animals were examined pre-operatively, and then three to four and seven to ten days post-operatively. A third examination was performed four weeks post operatively in the seropositive group, but not in the seronegative animals nor in 8730, as there were no significant inflammatory findings in the second examination of this group.

Inflammatory findings were graded on a scale of 0 – 4, as follows: 0 – no abnormalities detected; 1 –retinal petechia or vitreous hemorrhage; 2 – signs of mild anterior or posterior uveitis; 3 - focal retinal atrophy or detachment; 4 – significant uveitis or widespread ( > 15%) retinal atrophy or detachment. Findings that were potential complications of the injection (e.g., conjunctival hyperemia or focal cataract) are not presented.

N/A – examination not performed.

## Discussion

Pre-existing anti-AAV-NABs are likely to affect the safety and efficacy of IVT gene augmentation therapy if there is a straight-forward correlation between circulating NABs and NABs found in the vitreous. In this study of a naturally-occurring large animal model of *CNGA3* achromatopsia, we demonstrate a pre-operative anti-AAV2 seropositivity rate of 21.4% in our cohort of experimental sheep. Age had a significant effect on seropositivity, with animals older than one year of age having an approximately X7 higher risk of being seropositive than animals younger than one year of age. Seropositivity rate in our cohort was comparatively lower than that found in healthy human populations and in clinical trial patients, which ranged between 22-90% in different reports [7, 23, 24]. The effect of age on seropositivity is consistently reported both in the human population and in non-human primates [25, 26]. In humans, NABs are detected in newborns (presumably due to maternal antibodies), but their titers decline during the first year of life and subsequently rise and peak at the age of three years [27]. The effect of age is particularly important in treatment of inherited diseases that are diagnosed early in life, encouraging application of AAV based gene augmentation therapy within this time window, before natural exposure to AAV.

Neutralizing antibody titers increased following IVT AAV administration in 9/12 sheep in the current study (Table 1). The degree of serum neutralization increased significantly post-operatively in the seronegative group; however, there was no significant difference in the degree of post-operative serum neutralization between seronegative and seropositive groups. While there was no significant effect of previous exposure on post-operative NAB titers, only sheep from the seropositive group presented with clinical symptoms of inflammation at all post-operative examinations (Table 2), perhaps suggesting activation of other branches of the immune system in those sheep, such as the innate immune response [28, 29]. The inflammatory response could also be related to the injection itself rather than to the viral vector, similar to the sterile inflammation reported in response to intravitreally injected anti-VEGF agents [30]. Similarly to the results presented here, a recently published analysis of phase I/II clinical trial in patients with Leber hereditary optic neuropathy treated with IVT AAV2 injections did not find an association between post-operative serum NAB titers and the degree of ocular inflammation. However, in that report previous exposure to AAV was also not associated with a higher degree of clinical ocular inflammation [31]. In our study, no additional treatment, other than the topical neomycin and dexamethasone solution administered for a week (see Methods), was administered to any sheep. Therefore, the increase in number of seropositive animals with clinical signs noted in the second examination (Table 2) may be due to cessation of treatment. Likewise, the decrease in the number of seropositive animals with clinical signs noted in the third examination is due to self-resolution of the signs.

The humoral immune response against AAV consists of both NABs and binding antibodies (BABs). These non-neutralizing BABs, not measured in the current study, could also have an effect on safety and efficacy of AAV-based gene augmentation therapy, as they were previously shown to affect cellular transduction and tissue biodistribution [27, 32]. Moreover, it has been shown that both innate and adaptive cellular responses are triggered by intraocular AAV injection [33, 34]. These could likewise play a role in the pathogenesis of clinical signs of inflammation documented in our study. A recent study by Chan et al. demonstrates a strategy to overcome AAV activation of cellular immune response; in their study they engineered a vector that can evade activation of Toll-like Receptor 9 (TLR9), thereby reducing activation of both the innate and T-cell response and inflammation [35]. Further strategies of prevention and management of immune responses to intraocular viral vector administration are reviewed in Ren et al. [36].

Sheep in the current study were injected with AAV2.7m8 carrying one of three transgenes. While several previous studies report on possible toxic or inflammatory effects of different transgenes, and specifically GFP that was used in the current study [16, 37], in this study we were not able to demonstrate a significant effect of transgene type on the degree of immune response. This is most likely due to the small number of animals treated with each of the three transgenes, an important limitation of this work. The effect of the viral dose on immune and inflammatory responses is also often investigated. Higher viral doses most commonly result in an increased antibody response [8, 16, 38], but several studies present contradicting results, with either no effect of viral dose, or a higher immune response to lower doses of AAV [39–41]. In this study we did not observe a significant effect of viral dose on NAB titers, despite the 10-fold dose range we injected. The lack of significance in the current work could likewise be due to the small sample size.

Indeed, sample size is a major limitation of this study. Studies in large animal models such as the sheep are potentially more translatable to human patients, but they come at a cost of minimizing sample size. The limited sample size in this study also prevented us from investigating the effect of the number of eyes injected due to unequal representation of unilateral vs bilateral injections in both the seronegative and seropositive groups. Nevertheless, the number of injected eyes was to some degree expressed in the doses, since bilateral injections resulted in higher doses of virus injected.

Another important limitation of the current work is the use of a single serotype to detect NABs. Cross-reactivity between different AAV serotypes is known to occur [8] and therefore the presence of NABs directed at other serotypes could have affected the results presented in this report. A future, more comprehensive serological study, including analysis of both NABs and BABs directed at several AAV serotypes, is warranted. It is also worth noting that the seroprevalence against AAVs is influenced by the antibody assay used and the definition of seropositivity in different trials [42]. Cross reactivity between NABs against the different AAV serotypes should also be studied. Indeed, it is important for the scientific and medical communities to clearly define standards for seroprevalence analysis, and to determine whether there is a correlation between antibodies found in the blood and in the vitreous. Finally, because most of the vectors we injected in the seropositive sheep carried non-coding sequences, we were unable to test for correlation between serological response and gene expression.

The eye was historically considered to be an immune privileged site, as a result of several features. These include the blood-retinal barrier formed by tight junctions between endothelial and retinal pigmented epithelium cells as well as in retinal vessels, the lack of anatomically defined lymphatic drainage and an intraocular anti-inflammatory and immunomodulatory micro-environment [43]. Furthermore, wild-type AAV was historically considered as non-pathogenic and low-immunogenic [34]. However, it is becoming clear that the immune system *is* activated following intraocular viral vector delivery, a finding that has important implications for the safety and efficacy of therapeutic AAV-based gene augmentation therapy. The results presented here suggest that pre-existing anti-AAV NABs could contribute to an increased local inflammatory response following IVT delivery. Screening potential gene therapy patients for pre-existing anti-AAV NABs, as well as utilizing methods to overcome pre-existing immunity (reviewed in 29), are therefore of great importance, especially in gene augmentation treatment of systemic diseases. As clinical trials evaluating the safety and efficacy of rAAV-based gene augmentation therapy in *CNGA3* achromatopsia patients are ongoing, the study presented here may have implications for these trials as well as for other ocular gene augmentation therapy trials utilizing rAAV.

## Data Availability

The datasets generated during and/or analyzed during the current study are available from the corresponding author on reasonable request.
